# Ketogenic Diet Applied in Weight Reduction of Overweight and Obese Individuals with Progress Prediction by Use of the Modified Wishnofsky Equation

**DOI:** 10.3390/nu15040927

**Published:** 2023-02-12

**Authors:** Gordana Markovikj, Vesna Knights, Jasenka Gajdoš Kljusurić

**Affiliations:** 1 Faculty of Technology and Technical Sciences-Veles, University St. Kliment Ohridski-Bitola, Dimitar Vlahov bb, Veles 1400, North Macedonia; 2 Faculty of Food Technology and Biotechnology, University of Zagreb, Pierottijeva 6, Zagreb 10000, Croatia

**Keywords:** ketogenic diet, overweight, obesity, modified Wishnofsky equation, modelling

## Abstract

Ketogenic diet is often used as diet therapy for certain diseases, among other things, its positive effect related to weight loss is highlighted. Precisely because of the suggestion that KD can help with weight loss, visceral obesity, and appetite control, 100 respondents joined the weight loss program (of which 31% were men and 69% were women). The aforementioned respondents were interviewed in order to determine their eating habits, the amount of food consumed, and the time when they consume meals. Basic anthropometric data (body height, body mass, chest, waist, hips, biceps, and thigh circumferences) were also collected, in order to be able to monitor their progress during the different phases of the ketogenic diet. Important information is the expected body mass during the time frame of a certain keto diet phase. This information is important for the nutritionist, medical doctor, as well as for the participant in the reduced diet program; therefore, the model was developed that modified the original equation according to Wishnofsky. The results show that women lost an average of 22.7 kg (average number of days in the program 79.5), and for men the average weight loss was slightly higher, 29.7 kg (with an average of 76.8 days in the program). The prediction of expected body mass by the modified Wishnofsky’s equation was extremely well aligned with the experimental values, as shown by the Bland-Altman graph (bias for women 0.021 kg and −0.697 kg for men) and the coefficient of determination of 0.9903. The modification of the Wishnofsky equation further shed light on the importance of controlled energy reduction during the dietetic options of the ketogenic diet.

## 1. Introduction

In recent years, obesity became a serious global health crisis with prevalence increasing nearly threefold from 1975 to 2016 [1]. Research indicates the connection between obesity and numerous diseases and health complications, such as cardiovascular diseases, various types of cancer, type 2 diabetes, hypertension, polycystic ovary syndrome (PCOS), and many others [2,3]. It is important to emphasize that obesity can be prevented by establishing a balanced diet, adequate physical activity, and changes in behaviour and lifestyle [4]. Understanding the principles of energy balance is crucial [5] in approaching the global problem of the western countries: obesity. The concept of energy balance is based on the law of conservation of energy (energy conservation law: energy state of the organism = entered energy–expended energy), which states that energy cannot disappear or be created from nothing, but can only change its forms [6]. The source of energy in human diet are foods and drinks, with the main energy donors: carbohydrates, proteins, fats, and alcohol and the energy consumption varies throughout the day, but also throughout the lifespan [7].

Our organism strives for a state of energy balance and possesses regulatory mechanisms for this purpose. Regulation implies a complex physiological control system that includes neuronal and hormonal signals from the gastrointestinal tract, pancreas, and adipose tissue that reach the hypothalamus and the autonomic nervous system that innervates muscles, organs, and adipose tissue [5]. It was proven that this integrated regulatory system has stronger protection mechanisms for the loss of body mass than for the prevention of excess energy accumulation, and therefore there is a greater chance for the success of increasing body mass than reducing it [8]. The reduction in body mass is the result of a negative energy balance, i.e., increased energy consumption compared to intake [6]; however, sole reduction in energy intake does not result in continuous (infinite) and proportional loss of body mass. Reduction requires temporary changes in diet and physical activity, while long-term maintenance requires permanent changes, which seems to be more difficult [9] because studies show that 35 to 80% of individuals, who reduced at least 10% of their initial body mass, fail to maintain the reduced body mass for more than a year [10].

As successful reduction in body mass is classified, intentional loss of at least 10% of the original body mass is maintained at that level for at least one year. The criterion of 10% was set because already then the risk of diabetes and cardiovascular diseases was significantly reduced [11]. So, with the aim of a better understanding of an observed problem, models are developed, among which mathematical models were developed to try to understand the non-linearity of body mass loss during energy reduction as one of approaches in dealing with obesity. Numerous mathematical models were designed for the purpose of predicting body mass loss, which differ from each other according to the concept of how energy is stored and consumed [12]. The first such model, which combined all the knowledge about calories and energy metabolism developed for predicting the expected body mass based on the timeframe of energy intake reduction is the Wishnofski model from 1958 [13]. Doctor Max Wishnofsky researched energy from food, how it is stored in the body, and by what amount it is necessary to reduce energy intake in order to lose 1 kg of body mass [14]. He designed a regression model that was supposed to serve as a universal measure for assessing body mass change based on an energy intake reduction in a known time frame and with a caloric equivalent of one pound of lost or gained body mass of 3500 kcal (for 1 kg–approximately 7700 kcal) [12]:

(1) $$Weight\;\;loss\;\left[lb\right]=E_{s}\left[kcal/day\right]\cdot \frac{t\;\left[days\right]}{3500\;\left[\frac{kcal}{lb}\right]}$$

where:

*E_s_*—imposed daily deficit in energy stores (reduced energy intake or increased exercise generated energy output), [kcal/day];

*t*—duration of the diet [days].

Studies show that different diet patterns influence diet changes and maintain reduced body weight [2,15], and one of them is the ketogenic diet, which is characterized by a significant reduced intake of carbohydrates (<30 g/day) and standard protein intake (1.2–1.5 g/kg of ideal body weight or 1.0–1.2 g/kg of fat free mass) [16]. This diet is also often used in diet therapy of obesity, type 2 diabetes mellitus, migraines, polycystic ovary syndrome, and even epilepsy [17,18,19,20,21,22]. There are several types of eating patterns within the keto diet. A standard ketogenic diet implies that fats make up 70% of the daily energy intake (DEI), proteins 20%, and carbohydrates only 10%. In addition to the standard one, the cyclic ketogenic diet includes periods of carbohydrate compensation (after every 5 days the diet is followed by 2 days with increased carbohydrate intake), a targeted ketogenic diet that allows the addition of carbohydrates during periods of intense physical activity (25 to 50 g half an hour before training), and a high-protein ketogenic diet that is similar to the standard diet, but the macronutrient intake ratio is changed (fats: proteins: carbohydrates = 60:35:5) [23].

According to all of the above, the aim of this paper is to demonstrate the usefulness of the Wishnofsky equation based on collected data of people on a ketogenic diet. Several requirements were studied, the most important of which is the accuracy of predicting the course of body mass loss over a certain period of time, as well as different phases during the energy restriction and macronutrient intake based on the ketogenic diet guidelines.

## 2. Materials and Methods

In the study were included 100 healthy adults (31% of them are males) from Skopje, North Macedonia, enrolled in the program of weight reduction by following keto diet principles. Their anthropometric data (weight, height, circumferences of: chest, waist (two places: (i) narrowest part and (ii) at the navel region), hips, biceps, and thighs), diet habits before the diet, and some basic information related to their food intake were collected in an individual interview with a nutritionist. During the interview were collected such data as frequency of consumption of some nutrition dense food (fruit and vegetables) as well as caloric food (sweets, salty snacks, seeds, and nuts) and beverages (carbonated drinks vs water). The time of meal consumption was also recorded. The measurements were collected since April 2022. All respondents signed the agreement that their data can be used exclusively for scientific purposes, and the principles of the GDPR were respected.

Observed anthropometric parameters of the participants were collected following the recommendation of Casadei and Kiel [24] and they are given in Table 1.

In addition to the anthropometric parameters, for each subject, anamnesis was taken about the basic state of health, as well as the number and type of meals and the time of consumption. At the first medical examination, the subjects’ body mass, body height, and circumferences of arm, leg, waist, and hips were measured. The initial body mass index (BMI) was expressed as the ratio of the body mass to the square of the body height, and the target body mass for each subject was obtained in the range for the targeted normal BMI (20–25 kg/m^2^).

At each follow-up examination, subjects’ body mass and circumference were measured to monitor progress. In the case of adequate progress, the allowed energy intake is increased, i.e., the person moves to the next phase of the ketogenic diet. However, if at some point there is a stagnation of progress or an increase in body mass, the subjects are returned to the previous phase and their energy intake is reduced.

The Wishnofsky equation was used because it depends on the phase of the body mass reduction process, and was modified because during the ketogenic diet were included seven different phases (Table 2) and the average energy nutrient composition for the last phase is given in the supplementary Table S1 for menus created by a dietitian and medical doctor. 

According to the energy intake of different phases, the Wishnofsky equation (Equation (1)) was modified as follows:

(2) $$W\left(t\right)=W_{0}-0.454*\Delta EB*\frac{t}{3500}$$

(3) $$Wt_{j}=Wt_{j-1}-0.454*EB_{i}*\frac{t_{j}}{3500}$$

where

*W*_0_—initial body mass [kg]; *W(t)*—expected body mass [kg] after *t* days where the energy intake was reduced for *∆EB* (reduced daily energy intake [kcal/day] compared to the required one);

*Wt*_*j*−1_—initial body mass for the new ketogenic diet phase (*i* = 1, …, 7), the ketogenic diet phase (*i*) can be repeated several times (*j* = 1, …, n) and the last one ends when *Wt_j_* = *Wd* (desired body mass). When calculating *EB_i_*, the mean values of the energy range of the different phases of the ketogenic diet were used (Table 2).

**Figure 1:**
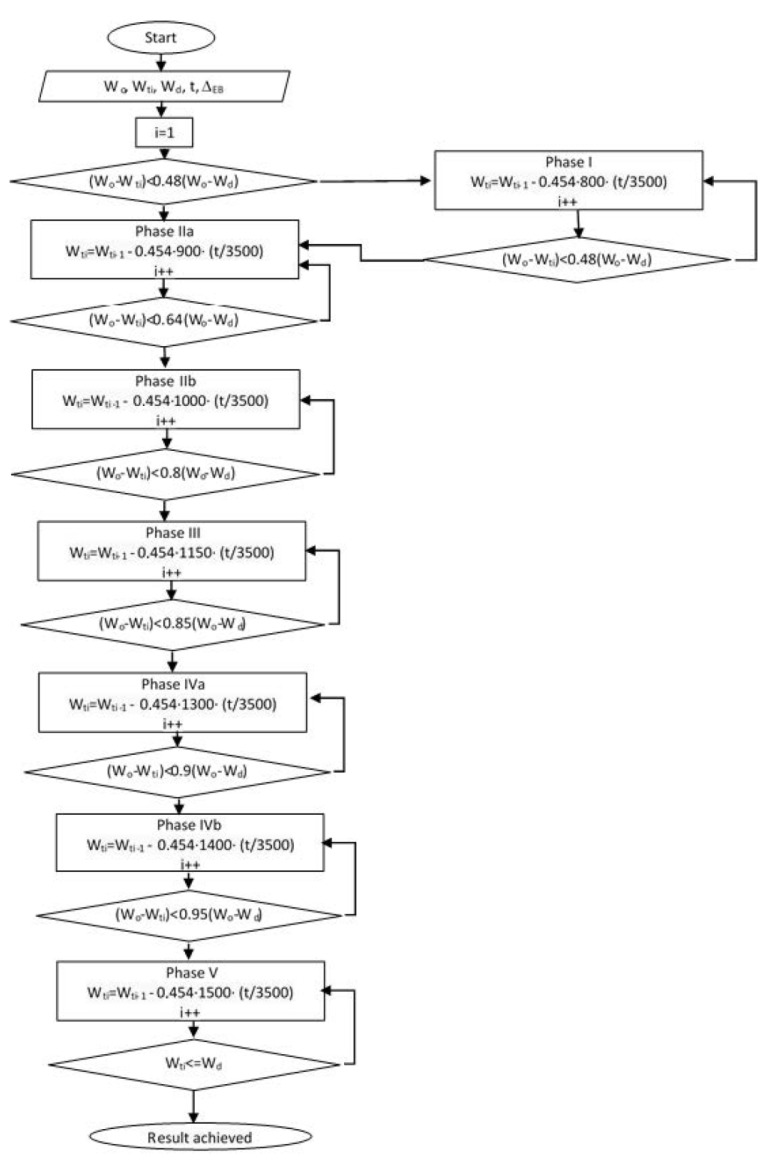
Flow chart presenting ketogenic diet phases.

The flow chart (Figure 1) presents the ketogenic diet implementation process from the initial body mass (*W*_0_) to the desired one (*Wd*). Patients start with 800 kcal in phase I until they reach 48% of the difference between initial body mass (*W*_0_) and the desired (*Wd*). After this phase, the energy intake is increased to 900 kcal (Phase IIa). By reaching 64% of the difference between initial body mass (*W*_0_) and the desired one (*Wd*), the daily energy intake is upgraded to 1000 kcal (IIb phase). In phase III, the patient reached less than 80% of the difference between *W*_0_ and *Wd,* and the daily calorie intake is than 1150 kcal. In phase IVa, the patient reached less than 85% of the difference (*W*_0_–*Wd*) with 1300 kcal per day. Phase IVb, starts when the patient reaches 90% of the difference between initial body mass and desired (*W*_0_–*Wd*) with 1400 kcal per day. The last phase (phase V) increases the energy intake to 1500 kcal when the achieved body mass is less the 5% from the desired one.

As variables are indicated values for body masses that were recorded for the patient after each examination at a certain diet phase, *Wt_j_*, *j* is the number of check controls, while other primary parameters are: *W*_0_ as the initial body weight, *Wd* as the desired body weight, and previously mentioned *EB* as intake of energy during the day. 

Patients who are over-weighted, but still not obese, and where the difference between the initial body mass and the desired one (*W*_0_–*Wd)* is less than 48%, the diet is directed immediately into the second phase of the diet (*i* = 2: Phase IIa). If this is not the case, the diet plan will start with the first phase. The third phase is approached when the patient reaches 80% of the desired weight loss. In the remaining stages, the patient loses the remaining 20% of body mass.

In addition to all measurements, the expected body mass during each examination was also calculated using the Wishnofsky equation (based on the body mass recorded at the previous examination, the number of days since the previous examination, and the energy intake in that phase). None of three variables used in the calculation are a constant; body mass differs each time, and energy intake also changes analogously, i.e., the phase of the ketogenic diet. The number of days since the previous examination is different for each person.

At the end of the research, the data were statistically processed and the actual situation and progress were compared with the prediction based on the Wishnofsky equation.

All calculations were conducted by use of MS Excel. Calculated were the (i) minimal and maximal values in the observed data set, (ii) standard measure of central tendency (mean, mode), and (iii) standard deviation (SD) as a measure of dispersion. Relative frequencies (as percentage) were used in the display of results related to the eating habits of people involved in the weight loss program. Box-whiskers plot was used to show the progress of body mass loss and the reduced body mass index. The Bland–Altman chart is used to show the effectiveness of predicting body mass using the modified Wishnofsky equation. A simple linear regression was used to show the agreement of body mass data in a certain phase of the ketogenic diet with the exact body mass measured during the regular examination.

## 3. Results

**Figure 2:**
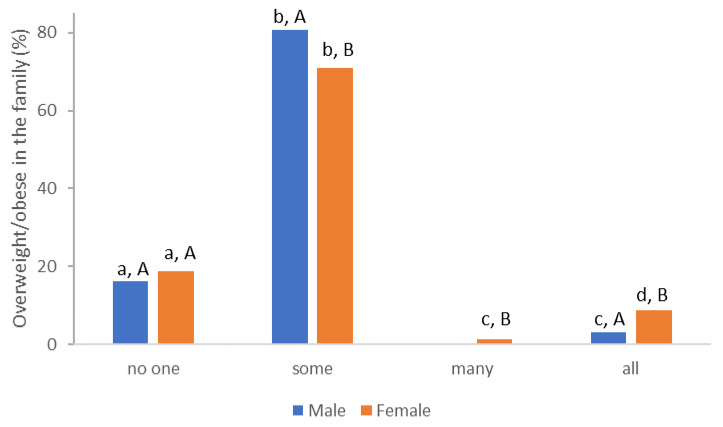
Prevalence of being overweight and/or obesity in the family (different lowercase letters: significant differences in the frequency of overweight or obese family members, within the same gender; different capital letters: significant difference in the frequency of overweight or obese family members within different gender).

During the first examination, an interview was conducted (with each subject) in which data were recorded on the frequency of overweight and/or obesity in the family (Figure 2), their eating habits, i.e., the frequency and time of eating (Table 3), and certain types of food (Table 4).

From the prevalence of obesity in the family, differences in the answers of the male and female population are visible; however, research by [25] Sattler and associates (2018) shows that it is weight-based stigmatization with motivation to exercise and physical activity in overweight individuals in connection with different genders. 

Information on the frequency of consumption of certain foods (sweet, salty, and seeds) and drinks was a source of information on the quality of eating habits (Table 3). Only one third of female and 48.39% female subjects consumed non or less than 50 g of sweets per week, while chips (including other salty snacks) were consumed by over 50% of subjects, regardless the gender is consuming in amounts less than 50 g/week. Unfortunately, it is a devastating fact that the amount of fruit and vegetables consumed during the week is limited to small amounts, indicating that energy-rich food, with low nutritional density, dominates their diet. Higher intake of fruits and vegetables increased weight loss [26]. In the investigated group, the frequency of consuming vegetables was significantly lower, although it can be consumed as a side dish, salad, etc. The following finding is related with the regional consumer habits, including high consumption of nuts and seeds. In the conversation during the interview, it was clear that seeds and nuts are consumed between meals in uncontrolled amounts, although the average caloric contribution in 100 g is in the range of 400–600 kcal [27]. 

Consumption of beverages shows an exceptional representation of carbonated beverages compared to water, which is consumed most often in the amount of 1–2 L in the male population (38.71%). Carbonated mineral water is also included in CO_2_ beverages. Over-weighted and obese individuals have higher demand on fluid intake, and improved hydration is a commonly used strategy by nutritionists to prevent overeating with the goal of promoting a healthy weight among patients [28]. It is a worrying fact that almost 56.52% of women and even 74.19% of men in the group of respondents do not consume water on a daily basis.

However, it is not only the combination of poor nutrition that is related to the problem of excessive body weight or obesity of the respondents, there is also the number of meals and their distribution during the day (Table 4).

In order to additionally determine the frequency of the most common number of meals in the respondents’ answers, the mode value was also calculated. Female subjects have more meals (mode value is 3 vs. 2 of the male population, respectively). Late meals dominate (second meal at around 4 pm) while the first meal is extremely late (regardless of gender, around 10 or 11 am) and a lot of them have late night meals (around midnight). 

The participants reduced their daily energy intake, guided by the ketogenic diet principles. Successful progress of the subjects can be seen in Table 5.

Such an approach in body weight reductions requires numerous examinations (6.8 for females and 8 for male subjects) and a long period of time in the program (79.5 and 76.8 for female and male subjects, respectively).

The reduction in all measured circumferences is dominantly in the waist and hip region for both genders (more than 20 cm reduction).

**Figure 3:**
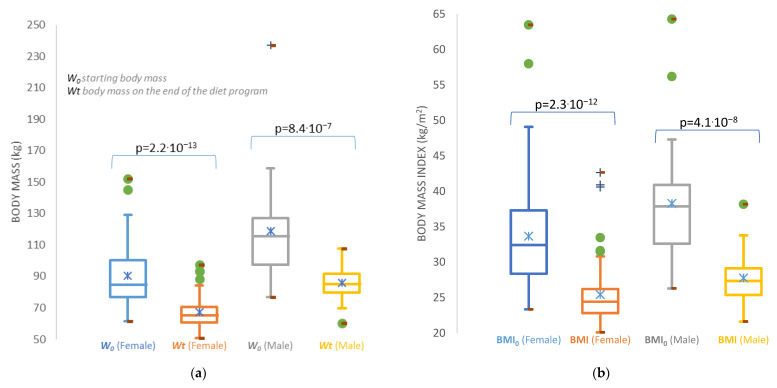
Box-whisker plots of: (**a**) reduced body masses of female and male subjects; (**b**) reduced BMI for female and male subjects enrolled in the ketogenic reduction diet program. Description of what is contained in the second panel.

Although the average body mass that was planned and achieved differs for both genders (Table 5), the success can be seen in Figure 3, indicating the achievement in body mass loss, as well as for the decrease in the body mass index. The first impression is that the male subjects failed to achieve the expected body mass index of normal nutrition. However, the male population more actively accepted physical activity, especially exercise, and therefore their body mass index is slightly higher due to an increase in muscle mass.

**Figure 4:**
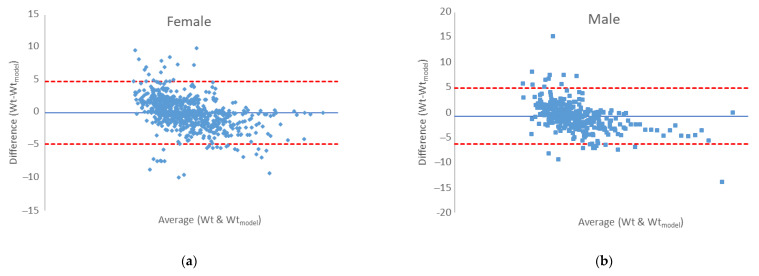
Bland–Altman plot for body mass of different phases of ketogenic diet for (**a**) female and (**b**) male subjects.

**Figure 5:**
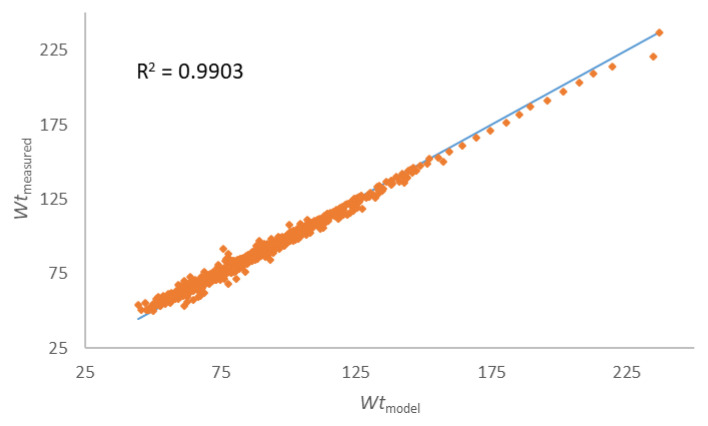
Compatibility of estimated body masses with the modified Wishnofsky equation (*Wt*) during the reduction ketogenic diet and established body masses at the end of the program.

An accurate perception of the expected body weight after a certain time of reduction in energy intake is necessary for people who are on a weight loss program, but also for nutritionists who lead the program in order to design the appropriate next step of the weight management program [29]. Therefore, last results are devoted to the efficiency of the modified Wishnofsky equation in predicting expected body mass after a certain phase of their diet. It is suggested to use correlations and regressions to assess the agreement between the two quantitative measurement methods, as in our case with the experimental values of body mass, and the predicted one by use of modified equation by Wishnofsky. Correlation will give an insight into the relationship between one variable and another, but will not indicate differences, and thus is not an ideal method for assessing comparability between methods. An alternative is the Bland–Altman graph, which as a basis for quantifying the agreement between two quantitative measurements offers the study of the relationship of the mean difference in the limits of agreement. The Bland–Altman graph defines intervals of agreement, and acceptable limits must be defined in advance, based on the set goals [30]. Our agreement is presented in Figure 4. For both genders, the bias values are very close to zero (−0.697 kg and 0.021 kg) for male and female measures, respectively. The error is 0.0614 in prediction of male body mass and 0.058 in predicting female body mass of a certain phase of the ketogenic diet. A certain proportion of outliers (Figure 4., dots outside the area of limits of agreement (±1.96 × SD)) is visible, which is dominantly the result of non-adherence to the principles of the keto diet, and precisely the difference between the expected body mass (>5%) vs. the measured body mass during the control examination, which is the indication of relapse. In the supplement material, Figure S1 shows the repetition of ketogenic diet phases for one relapsed participant who started the program from the beginning for three times. The disproportion between the expected body mass (calculated by the modified Wishnofsky’s equation) and the measured mass is evident, and greater than ±5%. Here, it must be emphasized that none of the input data of the respondents were taken as an outlier (precisely the extreme values, such as the body weight of 237 kg of a male person) that influence the increase in the error. For this reason, the regression line of the experimental values of body masses and those obtained by the modified model is shown (Figure 5).

The last efficient test is presented with the regression line of the body masses measured during the examinations and those predicted by the use of the modified equation of Wishnofsky (Figure 5), and it is clear that, even with outliers in the data set, there is still an extremely strong connection between the observed data (*R*^2^ = 0.9903).

## 4. Discussion

In order to avoid the yo-yo effect and preserve weight loss progress, Wing and Phelan [31] defined six key strategies that should be followed: (i) increased level of physical activity (1 h/day), (ii) change in eating habits in the context of avoiding energy-rich foods and foods rich in fats, (iii) regular breakfast (latest in 2 h after waking up), (iv) regular monitoring of body mass, (v) constant eating pattern, and (vi) reacting to minor mistakes by correcting them in a timely manner so that they do not cause a greater return of lost body mass and causing a negative impact on the weight loss progress. Theoretically, thebasic principle of losing weight is quite simple: spend more energy than you take in. However, while the fact is that we have to reduce our calorie intake, it is important to know the exact source and amount of calories eaten, and whether the body can be influenced in the tendency to lose and later to restore the balance. The primary “fuel” of the human body is glucose, i.e., carbohydrates. Therefore, when glucose stores are low, as is the case during a ketogenic diet, the central nervous system must find an alternative source of energy [4]. Then, the energy source becomes ketone bodies–acetoacetate, beta-hydroxybutyrate, and acetone. These molecules are the product of ketogenesis that takes place in the mitochondrial matrix in the liver. Under normal conditions, they are found in the body in very low concentrations (<0.3 mmol/L). Given that they are similar in structure to glucose, they have the ability to use a glucose transporter to cross the blood–brain barrier to be used as an energy source when they reach a concentration of 4 mmol/L in the body. The described state of elevated levels of ketone bodies in the body is called “ketosis” [32]. 

It is believed that this mechanism forces the body, due to the lack of glucose, i.e., carbohydrates in the diet, to consume fat reserves and thereby reduce the amount of fat tissue and total body mass. In addition, Ketone bodies serve as an alternative energy source for brain metabolism [33]. Bypassing the traditional ways of releasing energy through glycolysis in favour of using ketone bodies has a significant effect on the body, and although the entire mechanism is not fully understood, it is clear that bypassing the metabolism of carbohydrates in the brain can also lead to positive health effects, such as a reduced frequency of epileptic seizures [5,34].

The ketogenic diet guidelines show that the basis of the diet should be fats. Unsaturated fatty acids are allowed, such as nuts, seeds, avocado, tofu, and olive oil, but a higher intake of saturated fatty acids is emphasized, such as butter, animal fat, coconut oil, butter, etc. Proteins are the next macronutrient when considering the share of daily energy intake. There are no big differences in the recommendations of protein sources, but poultry meat, fish, and red meat are recommended in larger quantities than eggs, cheese and milk, and dairy products. In the end, carbohydrates remain [16,23]. As can be seen in our investigated group, the (i) time of consumption and (ii) the number of meals are also important issues related to being overweight. A study conducted among Japanese women showed that those who consumed late dinners or bedtime snacks were more likely to skip breakfast, which explains the late first meal of the investigated subjects. The same study concluded that having a late dinner or bedtime snack is associated with a higher probability of being overweight/obesity [35]. Low-carb vegetables are allowed, i.e., green leafy vegetables, broccoli, cauliflower, Brussels sprouts, asparagus, peppers, onions, garlic, cucumber, mushrooms, etc. In addition to vegetables, fruits contain a high proportion of carbohydrates, and therefore only berries are recommended [36]. Extensive literature overview in the meta-analysis conducted by Arnotti and Bamber [26] investigated the fruit and vegetable consumption in overweight or obese individuals (3719 participants), and it was shown that the effect was large (−2.81 kg; *p* < 0.001). Lipid metabolism, which is a key factor in planning body mass reduction, is an extremely complex process, and models are available that simulate its development with the aim of understanding its biological processes [37]. The models can also be used to optimize and define sustainable diet indicators where the ketogenic diet shows success [38,39]. In order to help both nutritionists and people who are on a weight loss program, a modified model of weight gain from the Wishnofsky equation was proposed. Having a perception of what to expect after a certain period of reduced energy intake is more than encouraging for participants in the weight loss program [25]. Decades after Wishnofsky’s equations, different mathematical models were created for predicting expected body mass in a certain time frame based on the law of conservation of energy, and those models differ according to differences in the understanding of what energy consumption entails and what the energy state of the organism entails [40]. The main requirement of a model is its simplicity and acceptable error [37,38,39]. Our results show that the prediction of expected body mass during the reduction keto diet using the modified Wishnofsky equation is extremely well aligned with the actual progress of people in the weight loss program, regardless of gender. The modification of the equation that includes changes in the phases of energy intake during the ketogenic diet is important because it is not a linear relation of body mass loss, but a non-linear process that is taken into account in this way. An extremely important factor is the time (t) of a certain phase of the diet in which a person establishes control over eating habits and continues with a further constant decrease in body weight. Deviations greater than 5.8% in women and 6.1% in men are an indicator of non-adherence to the basic principles of the diet, and are a corrective factor for the person on a diet and their nutritionist, because one of the goals is certainly the prevention of the “yo-yo” effect in the respondents. Thus, the so-called confidence interval values in the Bland–Altman graph will indicate the above and that the modified Wishnofsky equation did not successfully predict the expected body mass. This effect was confirmed in his research by Thomas and colleagues [13], who state that the use of the Wishnofsky’s equation is a rule that is easy to apply, but can lead to an error in predicting body mass loss; however, in the absence of simple and most understandable solutions, it is also an acceptable smaller error [39] in the expected value of body mass during the weight loss program.

As with each method, this model has also a disadvantage: it does not explain the metabolic adaptations that occur in the body, and it also does not take physical activity as an input in the calculations. The average BMI for the male population was slightly higher than 25 kg/m^2^, but according to the findings of Weber and co-worker [21], ketogenic diet helped in preserving muscle mass in patients with cancer, and the study of Pasiakos and coworkers [41] conducted on adults varying levels of dietary protein on body composition during energy deficit concluded that consuming dietary protein at levels exceeding recommendations may protect fat-free mass during short-term weight loss. The physical activity in obese people [25] will also affect the increase in muscle mass and consequently affect the BMI, although BMI does not distinguish muscle and fat mass. The focus is exclusively on the energy intake and the time frame of its reduction. Given the limited time period of this research, future research should include physical activity as well as the energy intake during the stabilization of energy intake after the restriction, because the potential of the model was confirmed also for those who had phases in which they returned to increased and inappropriate energy intake.

## 5. Conclusions

The implementation of supervised body weight loss, with the guidelines of the ketogenic diet, is primarily focused on the reduction in carbohydrates and energy. The proportion of body mass loss is dictated by the sequence of phases of the diet, and with medical supervision, the third phase (of 1300 kcal) of the diet occurs after 60–80% of the target body mass loss (*Wt-W*_0_). For the patient, cognition of the flow and duration of the diet itself is extremely important, and it is necessary to use tools such as prediction equations for body mass loss over a certain period of time. Body mass loss in different phases of the ketogenic diet can be effectively predicted by applying the equation by Wishnofsky, which represents a simple mathematical model that relatively accurately predicts the course of body mass change. It does not require a large number of input variables, which makes it useful for clinical practice, as well to monitor the progress and helping in creating an effective program for body weight loss, especially due to the large problem of obesity in the world.

Given that the modified model of Wishnofksy’s equation is proposed for predicting body mass reduction, taking into account that the person adheres to the prescribed guidelines and certain energy intake, the errors that occur are the result of the selection and procedures of the subjects, not the model itself. In this paper, an algorithm for the flow of the phases of the keto diet is generated. Following this algorithm leads to a reliable result of the desired weight. This is a good start for further research, as the next step would be to create a program that generates a variety of food and satisfies this algorithm model.

## Figures and Tables

**Table 1:** Basic anthropometric parameters of the subjects on the first medical examination.

Observed Parameters	Female (N = 69)		Male (N = 31)	
	Mean ± SD	[Min–Max]	Mean ± SD	[Min–Max]
Age (years)	37.5 ± 11.1	[18–68]	35 ± 11.8	[18–56]
Body height (cm)	163.8 ± 7.2	[147–184]	175.8 ± 7.4	[162–192]
Body mass (kg), W_0_	78.6 ± 17.2	[12.4–152.5]	103.3 ± 25.3	[60.3–237]
BMI (kg/m^2^)	32.9 ± 8.5*	[25.3–63.5]	38.3 ± 8*	[26.3–64.3]
Circumference (cm)				
Chest	88.4 ± 11.7	[64–136]	99.8 ± 14	[74–150]
Waist (narrowest part)	90.5 ± 12.9	[67–138]	101.8 ± 14.7	[74–162]
Waist (at the navel)	100.9 ± 13.5	[75–162]	110.7 ± 15.8	[80–164]
Hips	106.7 ± 13.2	[79–172]	114.9 ± 16.2	[80–179]
Biceps	33.8 ± 5.8	[9–62]	37.1 ± 4.1	[29–50]
Thighs	64.9 ± 9.1	[28–97]	68.5 ± 7.6	[56–97]

*: Statistically significant differences (*p* < 0.05); SD: standard deviation; BMI: body mass index; and W_0_: body mass on the beginning of diet program.

**Table 2:** Permissible ranges of energy intake with regard to the phase of the ketogenic diet.

Ketogenic Diet Phase (*i* = 1, …, 7)	Energy Range (*EB*), kcal (kJ)
I	750–850 (3140–3560)
IIa	850–950 (3560–3975)
IIb	950–1050 (3975–4395)
III	1100–1200 (4605–5025)
IVa	1300–1400 (5440–5860)
IVb	1350–1450 (5650–6070)
V	1500 (6280)

a, b-different energy levels of the same phase of the ketogenic diet.

**Table 3:** Frequency of consumption of certain foods and drinks.

	Consumption Per Week ^1^ (%)				
Sweets	non	<50 g	50–100 g	101–200 g	>200 g
Female	5.79 ^a^	26.09 ^a^	23.19 ^a^	37.68 ^a^	7.25 ^a^
Male	9.68 ^a^	38.71 ^b^	16.13 ^b^	16.13 ^b^	19.36 ^b^
**Chips**	**non**	**<50 g**	**50–100 g**	**101–200 g**	**>200 g**
Female	18.84 ^a^	33.33 ^a^	30.44 ^a^	13.04 ^a^	4.35 ^a^
Male	35.48 ^b^	25.81 ^b^	19.36 ^b^	9.68 ^a^	9.68 ^a^
**Vegetables**	**non**	**<500 g**	**500–1000 g**	**1001–1500 g**	**>1500 g**
Female	10.15 ^a^	88.41 ^a^	1.45 ^a^	0 ^a^	0 ^a^
Male	12.90 ^a^	87.10 ^a^	0 ^a^	0 ^a^	0 ^a^
**Fruits**	**non**	**<500 g**	**500–1000 g**	**1001–1500 g**	**>1500 g**
Female	17.39 ^a^	49.28 ^a^	18.84 ^a^	8.70 ^a^	5.80 ^a^
Male	19.35 ^a^	54.84 ^a^	16.13 ^a^	6.45 ^a^	3.23 ^a^
**Nuts**	**non**	**<50 g**	**50–100 g**	**101–200 g**	**200–500 g**
Female	26.09 ^a^	31.88 ^a^	13.04 a	26.09 ^a^	2.90 ^a^
Male	22.58 ^a^	32.26 ^a^	9.68 a	22.6 ^a^	12.90 ^b^
**Seeds**	**non**	**<500 g**	**500–1000 g**	**1001–1500 g**	**>1500 g**
Female	56.52 ^a^	21.74 ^a^	4.35 ^a^	17.39 ^a^	0 ^a^
Male	54.84 ^a^	22.58 ^a^	6.45 ^a^	16.13 ^a^	0 ^a^
**Carb. drinks**	**non**	**<0.5 L**	**0.5–1 L**	**1–2 L**	**>2 L**
Female	39.13 ^a^	28.99 ^a^	20.29 ^a^	7.25 ^a^	4.35 ^a^
Male	25.81 ^b^	12.91 ^b^	16,13 ^a^	38.71 ^b^	6.45 ^a^
**Water ^1^**	**non**	**<0.5 L**	**0.5–1 L**	**1–2 L**	**>2 L**
Female	56.52 ^a^	33.33 ^a^	10.14 ^a^	0 ^a^	0 ^a^
Male	74.19 ^b^	19.35 ^b^	6.45 ^a^	0 ^a^	0 ^a^

^1^ Per day; carb. drinks: carbonated drinks; different letters for investigated food group indicate significant differences for the observed consumed amount.

**Table 4:** Number of meals and their distribution during the day.

	Female (N = 69)		Male (N = 31)	
	Mean ± SD	Mode [Min–Max]	Mean ± SD	Mode [Min–Max]
No. of meals	2.8 ± 0.9	3 [1–6]	2.5 ± 1	2 [1–5]
Time of meal consumption (h) *				
1st meal	10.6 ± 2.5	10 [4.5–18.5]	11.3 ± 3.9	9 [3–21]
2nd meal	16.8 ± 2.4	17 [11–22]	15.9 ± 2.9	17 [9–21]
3rd meal	19.8 ± 2.6	21 [16–23]	19.5 ± 3.2	21 [13–24]
4th meal	21.3 ± 1.1	20 [20–23]	19.8 ± 0.5	20 [19,20]

* Time is expressed in the form 0–24 h; SD: standard deviation.

**Table 5:** Observed parameters on the end of the diet program.

Observed Parameters	Female (N = 69)		Male (N = 31)	
	Mean ± SD	[Min–Max]	Mean ± SD	[Min–Max]
Number of examinations	6.8 ± 4.6	[1–27]	8 ± 6.9	[1–38]
No. of days in the program	79.5 ± 171.6	[41–240]	76.8 ± 155.5	[10–247]
Planned body mass (kg)	64.3 ± 8.2	[52–97]	81.6 ± 12.6	[44–99]
Achieved body mass (kg)	76.7 ± 16.1	[50.7–146]	100.4 ± 24	[60.3–221]
Weight loss (kg)	22.7 ± 13.0	[5.6–55]	29.3 ± 12.6	[8.1–52.5]
BMI (kg/m^2^)	25.2 ± 3.8 *	[20.1–40.9]	27.8 ± 3.4 *	[21.6–38.2]
Circumference reduction (cm)				
Chest	19.9 ± 8.5	[2–40]	22.4 ± 10	[5–38]
Waist (narrowest part)	20.7 ± 9	[4–42]	24 ± 10	[8–50]
Waist (at the navel)	23 ± 9.9	[5–51]	26 ± 9.5	[8–41]
Hips	22.7 ± 10.9	[3–50]	26.3 ± 10.2	[8–50]
Biceps	9.3 ± 3.6	[2–17]	10.1 ± 2.8	[5–17]
Thighs	13.9 ± 5.2	[3–24]	11.6 ± 4.8	[4–24]

*: Statistically significant differences (*p* < 0.05); SD: standard deviation; and BMI: body mass index.

## Data Availability

The data that support the findings of this study are available from the corresponding author upon reasonable request.

## Supplementary Materials

The following supporting information can be downloaded at: https://www.mdpi.com/article/10.3390/nu15040927/s1, Figure S1: Trend of the expected body mass (BM) and measured body mass for a relapsed individual.; Table S1: Average energy and nutrient content of the 5th phase of the ketogenic diet.
