# Supplementary material for: Ketogenic Diet Applied in Weight Reduction of Overweight and Obese Individuals with Progress Prediction by Use of the Modified Wishnofsky Equation
Source: Nutrients. 2023 Feb 12;15(4):927. doi: 10.3390/nu15040927 (PMC9968058; doi:10.3390/nu15040927)
Supplement: Supplementary file 1 [file nutrients-15-00927-s001.zip › nutrients-2164145-supplementary.pdf]

Table S1. Average energy and nutrient content of the 5th phase of the ketogenic diet.

observed	Amount
Energy (kcal/kJ)	1500 / 6280 ± 50 / 210
Protein (g)[1]	136 ± 5.6
Carbohydrates (g)[1]	18.4 ± 1.1
Fats (g)[1]	95 ±4.3
Magnesium (mg)	360 ± 1.9
Sodium (mg)	1200 ± 44.2
Potassium (mg)	1200 ± 48.3
Calcium (mg)	360 ± 17.8
Magnesium (mg)	200 ± 15.5
Vitamin C (mg)	60 ± 2.7
Niacin (mg NE)	18 ± 0.8
Vitamin E (mg)	7.4 ± 0.4
Pantothenic acid (mg)	6 ± 0.3
Zinc (mg)	10 ± 0.9
Iron (mg)	8 ± 2.1
Vitamin B_6 (mg)	2 ± 0.08
Riboflavin (mg)	1.6 ± 0.03
Thiamine (mg)	1.4 ± 0.02
Cupper (mg)	1 ± 0.01
Manganese (mg)	1 ± 0.03
Vitamin A (μg RE)	800 ± 32.7
Folic acid (μg)	200 ± 10.4
Biotin (μg)	150 ± 6.2
Iodine (μg)	100 ± 4.2
Vitamin K (μg)	30 ± 1,1
Chromium (μg)	25 ± 0.9
Molybdenum (μg)	25 ± 1.1
Selenium (μg)	25 ± 1.3
Vitamin D (μg)	5 ± 0.25
Vitamin B_{12} (μg)	1 ± 0.2
Omega-3 (mg)	1000 ± 30

[1] share of the macronutrients followed the recommendations [23] (fats: proteins: carbohydrates = 60:35:5)

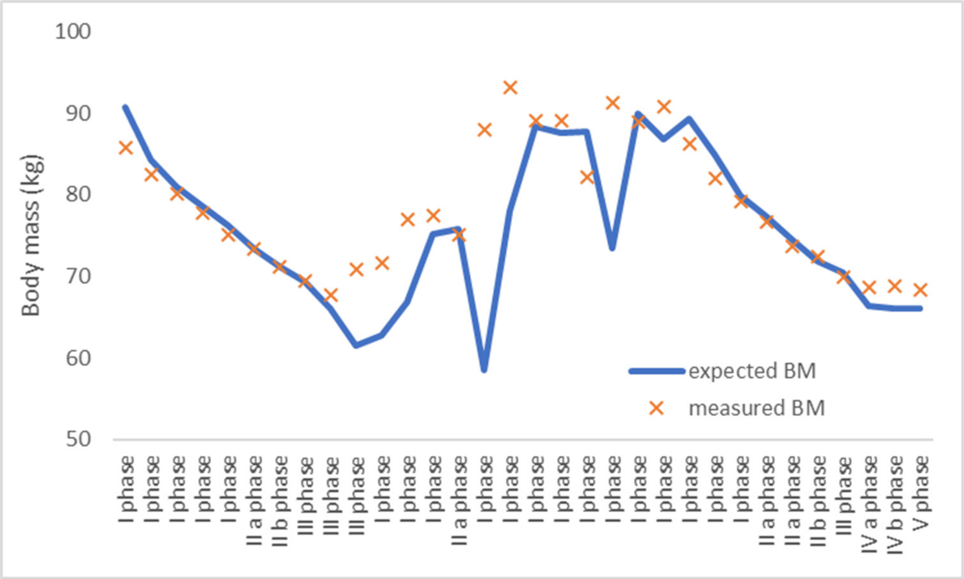

Figure S1. Trend of the expected body mass (BM) and measured body mass for a relapsed individual.
